# Indigenous and local communities can boost seed supply in the UN decade on ecosystem restoration

**DOI:** 10.1007/s13280-021-01593-z

**Published:** 2021-07-06

**Authors:** Danilo Urzedo, Simone Pedrini, Daniel L. M. Vieira, Alexandre B. Sampaio, Bruna D. F. Souza, Eduardo Malta Campos-Filho, Fatima C. M. Piña-Rodrigues, Isabel B. Schmidt, Rodrigo G. P. Junqueira, Kingsley Dixon

**Affiliations:** 1 ARC Centre for Mine Site Restoration, Curtin University, Perth, WA 6102 Australia; 2 Department of Sociology, University of Cambridge, Cambridge, CB2 1SB UK; 3 Empresa Brasileira de Pesquisa Agropecuária Recursos Genéticos e Biotecnologia, Brasília, DF 70770-917 Brazil; 4 ICMBio - Instituto Chico Mendes de Conservação da Biodiversidade, CBC - Centro de Avaliação da Biodiversidade, Pesquisa e Conservacão do Cerrado, Lago Sul, Brasília, DF Brazil; 5 Xingu Seed Network, Canarana, MT 78640 Brazil; 6 Instituto Socioambiental, São Paulo, SP 01238-001 Brazil; 7 Environmental Science Department, Federal University of Sao Carlos campus de Sorocaba, Sorocaba, SP 18052-780 Brazil; 8 Ecology Department, University of Brasília, Brasília, DF 70910-900 Brazil

**Keywords:** Community participation, Environmental justice, Local livelihoods, Native seed, Restoration economy

## Abstract

The UN Decade of Ecosystem Restoration is poised to trigger the recovery of ecosystem services and transform structural injustices across the world in a way unparalleled in human history. The inclusion of diverse Indigenous and local communities to co-create robust native seed supply systems is the backbone to achieve the goals for the Decade. Here we show how community-based organizations have co-developed native seed supply strategies for landscape restoration from the bottom-up. We draw on the interconnections over two decades of seed networks in Brazil and the emerging Indigenous participation in native seed production in Australia. From an environmental justice perspective, we provide a participatory seed supply approach for local engagement, noting local geographical, social and cultural contexts. Meeting large-scale restoration goals requires the connection between local seed production and collaborative platforms to negotiate roles, rights and responsibilities between stakeholders. An enduring native seed supply must include a diversity of voices and autonomy of community groups that builds equitable participation in social, economic, and environmental benefits.

## Introduction

Global policies and commitments are driving multiple incentives to restore hundreds of millions of hectares of degraded lands by 2030 (Chazdon et al. 2017). The Decade of Ecosystem Restoration (2021–2030) is poised to trigger the recovery of degraded ecosystems and create socioeconomic opportunities across the world (UN 2020). Following the 2020 bushfire crisis, most notably in Australia, increasing tropical deforestation, and the COVID-19 pandemic, global social movements are emerging to denounce how multiple crises exacerbate ongoing environmental injustices (Martin et al. 2020). Indigenous and local communities have demanded equitable landscape restoration measures through participation in political decisions and the creation of tangible local benefits (Reyes-García et al. 2019).

The translation of these ambitious policies and targets into real-world actions often fails to support local capabilities, enhance community livelihoods and deliver a net improvement in ecological functionality (Holl and Brancalion 2020). A key impediment in achieving the local to global restoration opportunity is the shortage of high-quality native seeds (Merritt and Dixon 2011; Jalonen et al. 2017). Native seed scarcity is often a result of the poor performance of the restoration market to keep enduring demands (Camhi et al. 2019), the limited application of knowledge and technologies (Pedrini et al. 2020), and restricted local participation in political decision-making processes (Nyoka et al. 2014; de Urzedo et al. 2019). Although local knowledge and engagement are considered key components to achieving successful projects, millions of Indigenous and local communities are commonly left behind in negotiations and planning of large-scale restoration programs across the globe (Erbaugh et al. 2020). These critical barriers accentuate the global use of tree planting as the surrogate for ‘ecosystem restoration’ where species are limited in diversity that focuses on a few, often non-native commercial varieties where the supply chain is dominated by a few large companies (Lewis et al. 2019).

Improving the availability of diverse native seeds in restoration has required the rapid development of applied strategies, techniques and technologies to solve complex on-ground issues for strengthening the seed supply chain (Pedrini and Dixon 2020). With an emergent restoration economy creating the demand, multiple stakeholders have structured commercial arrangements to scale-up plant material production—including seed, tubestock, rootstocks and cuttings to meet the large-scale restoration demand (Smith 2017; Atkinson et al. 2018). Conventional native seed enterprises, however, lack socioeconomic opportunities for local communities. In key restoration markets in Australia and Brazil, for instance, plant material markets are dominated by private companies which create few jobs, poor community engagement with few opportunities for enduring local livelihoods (Silva et al. 2016; Hancock et al. 2020). Multilevel restoration policies and financial incentives now need to consider alternative approaches to promote recognition and inclusion of local communities, so that grassroots actions address ongoing social and environmental crises (Ceccon et al. 2020).

Emerging community-based seed networks have connected local communities with multiple stakeholders to influence higher levels of governance for co-managing and innovating the seed supply chain from *source to sink* (Smith 2017; Schmidt et al. 2019; Piña-Rodrigues et al. 2021). Here we examine the co-creation of native seed networks for landscape restoration through participatory practices underpinned by local institutions and knowledge as an environmental justice intervention. We provide guidelines to community-based native seed supply, taking into account region-specific conditions and focusing on two decades of activities of the Brazilian seed networks operated through Indigenous and rural collectors in the Amazon, Cerrado, and Atlantic Forest, and emerging Aboriginal engagement with native seed production in Australia.

## The need for inclusive restoration approaches

**Fig. 1:**
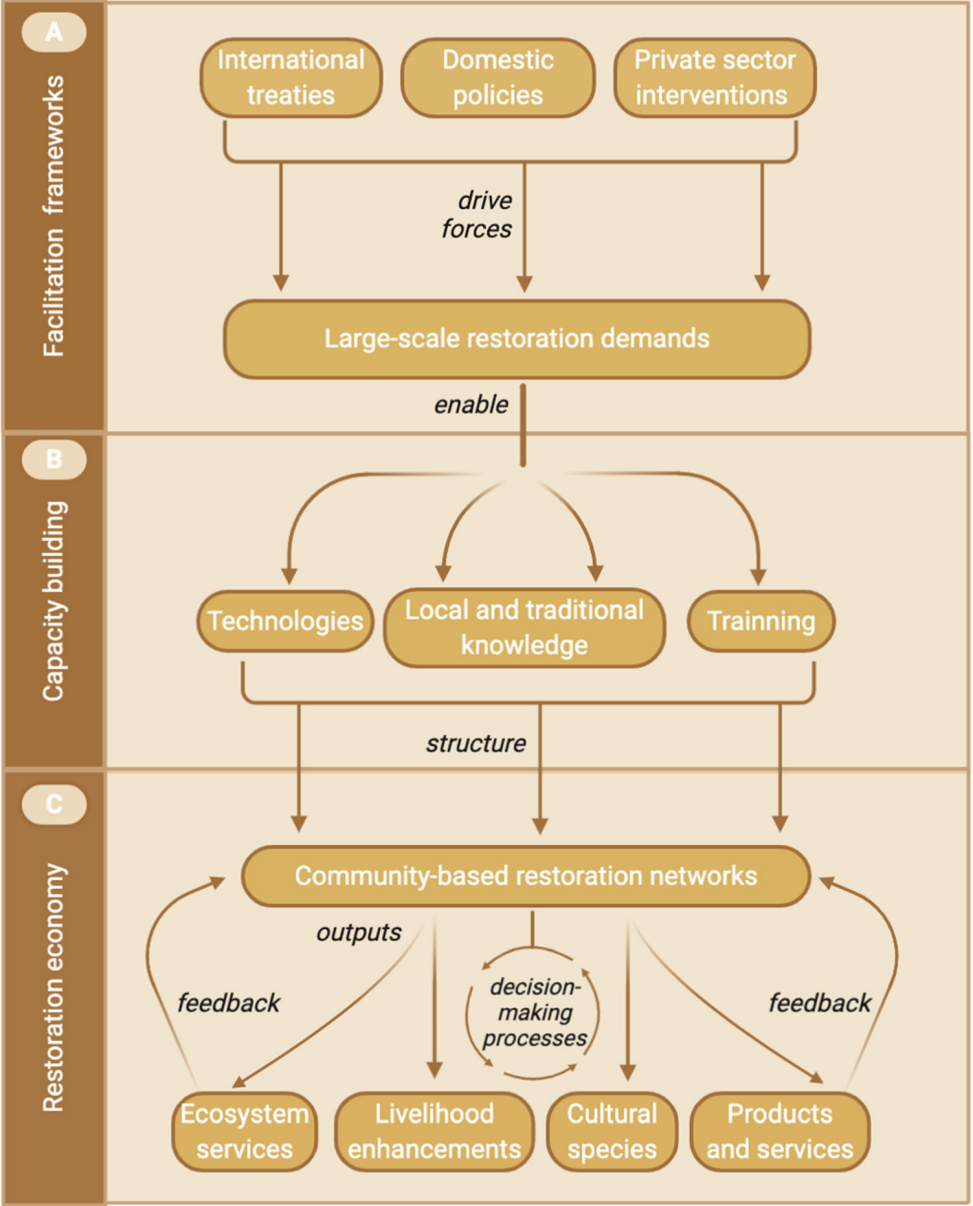
The emerging global restoration economy proposed for the Decade on Ecosystem Restoration. **A** Establishing large-scale restoration demand through multilevel political and financial investments driven by multilateral organizations, national and sub-national governments and private sector; **B** Enabling the interconnections between local and traditional knowledge with science and technology to create capacity-building processes that results in innovate restoration interventions, such as the native seed networks; **C** Structuring regional and local supply chain systems which are expected to create opportunities for Indigenous and rural communities to co-design and co-manage the supply of multiple products and services, including native seeds, which can improve human well-being across scales

The strategy of The UN decade draws on new trajectories to interconnect three pathways: building a global movement, generating political support, and developing technical capacity (UN 2020). International instruments are expected to drive political incentives for mobilizing social-ecological connections (Fig. 1) to combine domestic policies and private sector’s arrangements with community participation to scale up large-scale restoration. International initiatives and national programs have defined large-scale restoration pledges and incentives to drive efforts to restore 350 million hectares of degraded lands by 2030, such as the Bonn Challenge (IUCN 2011) and the Paris Agreement (UNFCCC 2015).

Amongst developing countries, Brazil has established one of the most ambitious restoration targets to restore 12 million hectares of degraded lands by 2030 (Brazil 2017). Brazil’s restoration pledge relies on mandatory forest restoration on private lands required by the Forest Code (Law N^o^ 12,65/2012). Implementing this national restoration goal would require an unprecedented effort to structure a restoration production network (Brancalion et al. 2019), including the estimate for 57 000 collectors to supply a total of 15 600 tonnes of native seeds during the UN Decade (de Urzedo et al. 2020). In contrast, the leading restoration drivers in Australia are mine site closure plans, Indigenous land management, land care programs (Hancock et al. 2020) and, more recently, carbon sequestration planting programs. The mining industry, in particular, must restore more than 300 thousand hectares of degraded lands throughout Australia, which will require a minimum of 1200 tonnes of seeds from diverse native species with ongoing annual demand likely for many decades for infill plantings (Merritt and Dixon 2011). Moreover, the Australian government has recently committed AUD200 million in response to the 2019–2020 bushfires to implement a national restoration program, including AUD5 million investment to develop a domestic native seed industry (DAWE 2020).

Although community participation is a critical component of successful restoration programs (Xu et al. 2012), global restoration policies and domestic initiatives fail to include effective mechanisms that would allow for the inclusion, engagement and benefit of local groups (Romijn et al. 2019). Legal and technical frameworks have limited the participatory planning and excluded Indigenous and community engagement to directly influence restoration negotiations and actions in Australia (O’Faircheallaigh and Lawrence 2019) and Brazil (de Urzedo et al. 2019). A transformative restoration economy must recognize and engage deeply with environmental justice values. Environmental justice emphasizes not only the necessity for access and distribution of environmental benefits (Schlosberg and Carruthers 2010), but also the challenges of addressing structural inequalities in power dynamics rooted in racial, ethnic, social class, age and gender discrimination (Martin et al. 2020). Meaningful ecosystem restoration actions should recognize the diversity of local members, equity in the distribution of benefits and risks, while creating multiple instruments that ensure vulnerable groups regain ownership and power in different political processes (Schlosberg 2004).

## Moving towards successful community-based seed production

From Latin America to Australasia, Indigenous peoples have been collecting, processing and selecting seeds for thousands of years to produce a diversity of products, including food, medicinal components and material culture (e.g., Lévis-Strauss 1952; Cane 1987). Local groups also have the innovation potential based on profound time experiences in local ecosystems. In recent decades, many seed programs worldwide have recognised these historical gaps and connected local communities to restoration markets built upon fair-trading schemes (Nyoka et al. 2014; Schmidt et al. 2019). Regional seed networks have united a diverse group of collectors who have collected, processed, and stored seeds with seed users and practitioners who implement different land restoration projects (Atkinson et al. 2018). Community seed networks are grounded in meaningful relationships and knowledge exchange between stakeholders, including universities, non-governmental organizations (NGOs), public and private sectors (Abizaid et al. 2016).

In Brazil, more than 24 networks have co-create opportunities to Indigenous and rural communities to supply native plant material to restoration markets over the last two decades (Piña-Rodrigues et al. 2021). In Australia, the emerging restoration economy ignites participation by Traditional Owners to create seed supply systems as ‘on country’ socioeconomic opportunities. We draw on seven well-structured community networks in these two countries to collect participatory-based experiences to co-development successful native seed networks for restoration alignment with the creation of socioeconomic benefits for local groups (Table 1). Although each initiative has established a specific set of interventions shaped by local context and particular stakeholder perspectives, there are essential values to be considered to operate native seed production as an opportunity to combine the restoration of ecosystem services with environmental justice.

**Table 1:** Key case studies of community-based native seed supply for landscape restoration in Australia and Brazil

Seed network	Country	Region	Ecosystems	Establishment (year)	Main restoration market
Gelganyem seed program	Australia	Western Australia	Tropical grasslands	2019	Mine site restoration
MEEDAC native seed farm	Australia	Western Australia	Dry shrublands	2018	Mine site restoration
Rio Tinto’s Weipa bauxite mine	Australia	Northern Australia	Tropical woodlands	2010	Mine site restoration
Cerrado de Pé Association	Brazil	Brazil Central	Tropical savanna	2012	Environmental offset
Jirau hydroelectric dam	Brazil	Western Amazon	Tropical rainforest	2011	Environmental offset
Vale do Ribeira network	Brazil	Southeastern Atlantic Forest	Tropical rainforest	2017	Restoration on private proprieties
Xingu Seed Network	Brazil	Southeastern Amazon	Tropical rainforest and Savanna	2007	Restoration on private proprieties and Environmental offset

## Key steps for community engagement with native seed supply

The following are key guidance steps to co-design and co-manage native plant material production that acknowledges issues of recognition, participation, and equity between participants (Box 1). Each of these issues is fully explored in the following subsections.

Box 1: Questions of relevance about community participation in native seed production for the restoration economy

- How is seed supply capacity co-designed to include local perspectives, interest and knowledge?
- Is the emerging restoration economy structured to promote culturally appropriate local participation based on ensuring long-lasting native seed demand?
- Who’s-who in the decision-making process of native seed production and commercialization?
- How are local knowledge and practices leading the capacity-building processes across sociocultural groups?
- Are community seed suppliers benefiting from the restoration environmental outcomes?

### Co-development of local supply capabilities

The first efforts to establish seed networks are commonly driven outside communities. Local communities can have high species ecological knowledge, but they frequently lack access to technical information on legal requirements and market opportunities for participating in seed supply for restoration (Brancalion et al. 2012; Silva et al. 2016). Participatory approaches for activating community-based systems rely on meaningful and long-lasting relationships between multiple stakeholders, including seed suppliers and users (Valette et al. 2020). Organizations with expertise in ecosystem restoration—such as government agencies, NGOs, and universities—play a crucial role in identifying seed market demands or stimulating restoration projects (Richards et al. 2015). Participation of local organizations increases trust and recognition to provide technical assistance and local engagement for the social licence to operate.

In Australia, partnerships between mining companies and Indigenous groups have resulted in many extractive resources agreements where operations are on traditional land (Bauman and Glick 2012). Although the social licence to close a mine requires a set of safeguards to protect Indigenous interests, overall mine closure plans have resulted in environmental injustices for Indigenous peoples (O’Faircheallaigh and Lawrence 2019). In response, emerging partnerships between the mineral industry, government agencies, research institutes and developmental program providers have developed socioeconomic opportunities for Traditional Owners through community-based seed supply systems for mine site restoration. Examples include the seed collection program of the Rio Tinto’s Weipa bauxite mine in Cape York Peninsula and the Gelganyem seed program for the closure of the Argyle Diamond Mine in the East Kimberley region of Western Australia.

The process of engagement requires carefully structured engagement planning. Communities are commonly contacted early in the process to identify local motivational triggers to stimulate and nurture local interest in engaging with seed production activities (de Urzedo et al. 2020). Prioritizing communities with well-established social organization and groups with the greatest need to access financial support build regional capacity to provide templates for engaging other less coordinated communities (Urzedo et al. 2016). In the Madeira river region of the Amazon, research institutes and NGOs supported community members of the local rural cooperative to connect their experiences with technical restoration approaches to meet emerging restoration demands for offsetting the impacts of the Jirau hydropower plant. In turn, the energy company contracted 81 members of the local cooperative for supplying native plants and planting services to restore 3000 ha of degraded lands (Kishy et al. 2020).

To ensure success in a community seed engagement program it is preferable an initial focus on just a few reference seed production groups with a modest seed demand focussing on well-known species that avoid seed production complexities (Campos-Filho et al. 2013). Once communities have defined what native species they are interested in supplying, external experts play an essential role in ensuring proper taxonomic identification to establish a production planning. Local groups can then map the seed collection areas, identify species, estimate the number of parent plants, and improve seed collection and processing techniques (Kindt et al. 2006). These elements are the starting point in structuring the community network’s production side, which then feeds into engagement, agreements, and co-management processes to effectively operate seed supply planning, actions, and assessments.

### Operating community business

There are different business arrangements for structuring seed supply chains that engage with local communities: hiring individuals and contracting local cooperatives (Brancalion et al. 2012; Smith 2017) or building a community-based business through a regional restoration market demand (Schmidt et al. 2019). Here, we present the operations of the community network model for seed marketing. This business model is a result of a 15 years’ experience of the Xingu Seed Network in the Amazon, one of the largest community-based native seed suppliers in terms of number of participants and seed volume for land restoration in the world (de Urzedo et al. 2020).

**Fig. 2:**
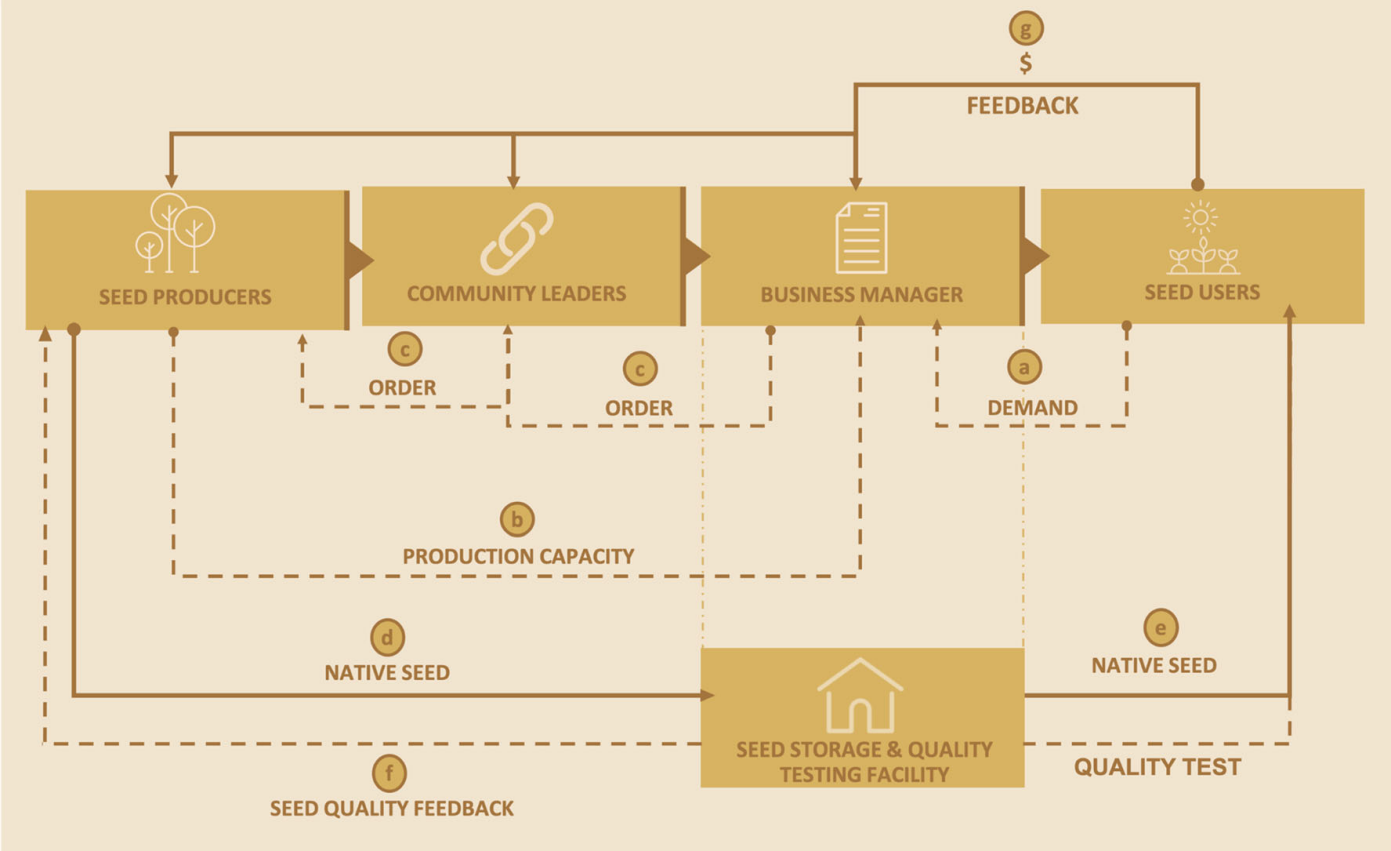
Commercial seed supply arrangements connecting different stakeholders in a flux of information (dotted lines) and resources (continuous line): (a) Native seed demand is identified and commercial contracts are established at the regional level; (b) producers plan their seed supply capacity; (c) a business office distribute the commercial seed demand between community groups according to their production capacity; (d) communities organize, produce and transport the seed batches to a storage and quality testing facility; (e) the seed business office assess and maintain the seed quality and then deliver the lots to users at the right time; (f) seed producers get the information about their seed quality; (g) consumers pay for the seed and provide their feedback to improve the supply chain

The Xingu Seed Network engages 600 Indigenous, rural and urban collectors who supply up to 25 tonnes of native seed yearly from 220 native species in the southeastern Amazon. The business involves many stakeholders geographically distant, requiring effective communication to select the right native seed in the right place and at the right time (Fig. 2). A centralized business coordination identifies the regional market demand, and coordinates the distribution of seed production between communities’ groups, ensuring yearly collection and income opportunities. Meanwhile, seed collectors observe in-field plant phenology in the seed collection areas to define their yearly seed production capacity (Fig. 2, part b) (Pedrini et al. 2020). With this local level estimate, the management office is responsible for matching collectors’ supply capacity with the total commercial seed demand of different projects. A significant factor in building community trust is the establishment of an annual minimum seed demand that ensures producers have clear financial expectations, suitable time commitments, with broad community involvement.

When the seed order list arrives in each community (Fig. 2, part c), local leaders are responsible for distributing the seed collection according to local agreements. A set of practices is adopted and adapted by groups, households, or individuals for seed collection, processing, and storage according to the local knowledge and access to infrastructure (Kindt et al. 2006). When the community complete the production activities, they deliver the seed lots—including labels—to a storage and quality assurance facility (De Vitis et al. 2020). Seeds are tested for quality to ensure all aspects from collecting time, cleaning and handling procedures result in the best quality seed (Frischie et al. 2020). Information on seed purity, viability and germinability need to be recorded and provided to the restoration practitioners to calibrate seeding density to optimise in-field outcomes (Pedrini and Dixon 2020). Collectors also apply simple seed quality and purity tests to revise and improve their management techniques.

**Fig. 3:**
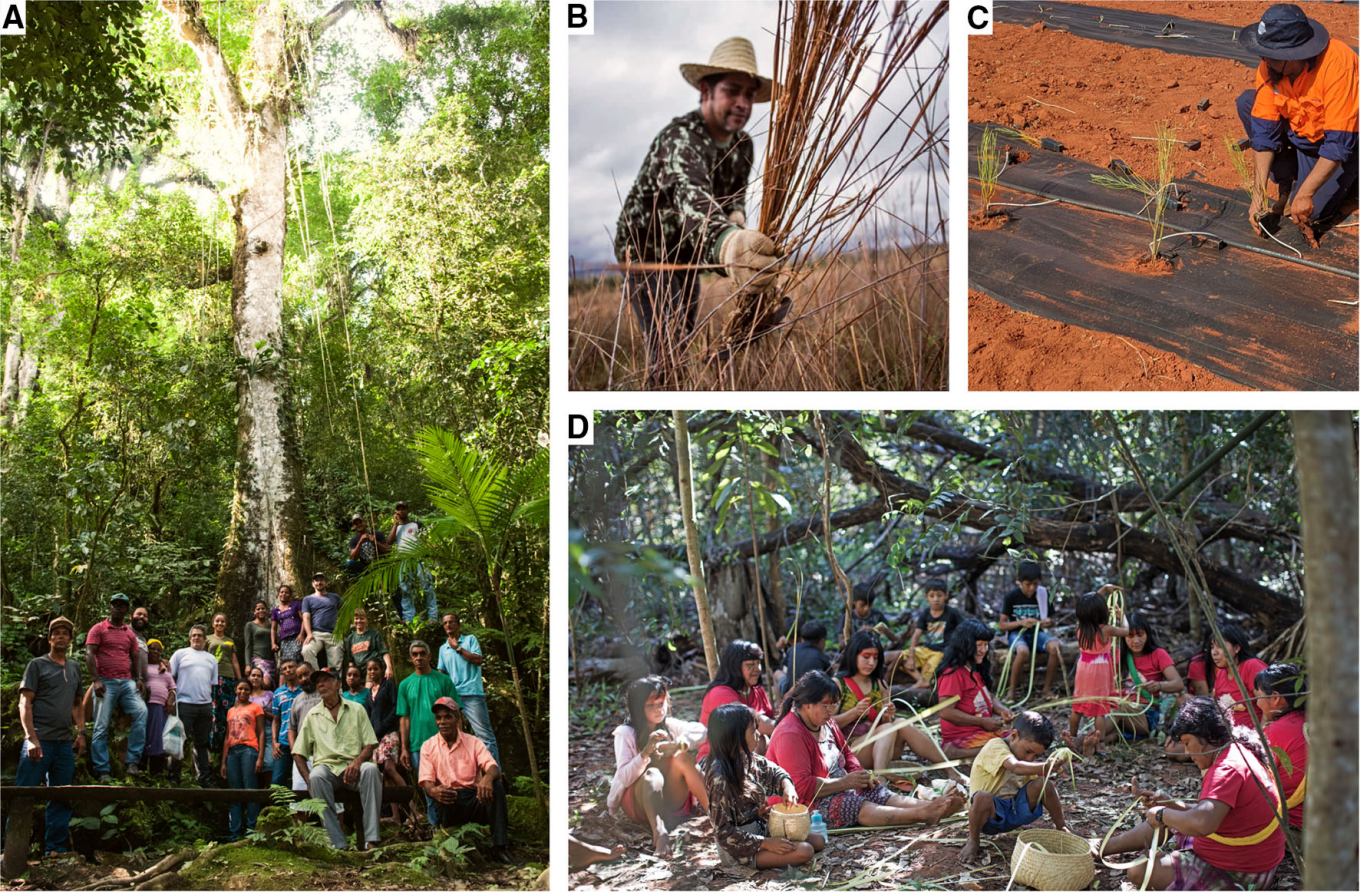
Community-based native seed supply activities, including **A** A community workshop with seed collectors and practitioners in Vale do Ribeira, Brazil’s Atlantic Forest (Claudio Tavares/ISA); **B** Native seed grass collection in the Neotropical Savanna of the Central-West region of Brazil (Tui Anandi); **C** Installation of an Indigenous owned and operated native seed farm in Morawa, Western Australia (Simone Pedrini); **D** Yarang women’s movement processing native seed in the Xingu Indigenous Territory, Brazilian Amazon (Carol Quintanilha/ISA)

The search for commercial partners is a continuous processes with a focus on establishing medium to long-term supply contracts with restoration markets. Supply agreements require shifting from a common logic of ordering seed at the beginning of a planting season to a long-term planning perspective where lead-times are sufficient to allow for cooperative planning, respect seed production phenology and critical uncertain climate scenarios (Broadhurst et al. 2016). Customers have learned that seed networks are not an on-demand enterprise but a community-based organization where market demand directly impacts community livelihoods. The business managers may be responsible for species selections or even for implementing the restoration project based on regional successful restoration models and on customer’s wishes (Fig. 2, part a). This support encourages the customers to purchase seeds and be confident to meet their restoration goals. Currently, the Xingu Seed Network’s business management roles are transitioning from a entirely external technical team to the inclusion of community members in order to promote local leadership not just for seed collection, but the entire supply chain arrangements (Fig. 3).

### Local institutional development

Community networks rely on complex relationships between multiple stakeholders responsible for exchanging skills, knowledge, and resources to operate the seed production chains (Valette et al. 2020). Coordinating the various interactions among local members and stakeholders outside the communities requires clear understanding of roles, rights, and responsibilities (Atkinson et al. 2018). Institutions refer to the social norms, patterns of behaviour, and agreements, either formal or informal mechanisms, which are influenced by the ways that stakeholders are participating in decision-making processes (Dequech 2009). Collective agreements define values and principles to establish platforms where members can discuss and negotiate at the local level based on participatory principles (Ostrom 2009). The cooperation between multiple stakeholders to co-manage seed networks lead to larger production capacity aligned with the inclusion of a greater diversity of species (Abizaid et al. 2016). These local institutions allow for a dynamic and organic evolution of the seed production procedures according to local needs, while a broader network outside the community is responsible for consolidating the market arrangement and other external resources (Urzedo et al. 2020).

At the community level, institutions assist local organizational processes to self-organize management systems among users to sustain the resources while respecting sociocultural norms (Ostrom 2009). The institutional structures in a seed network rely on the local agreements across multiple community groups for coordinating information sharing, conflict management, and political decision-making processes (Sanches et al. 2021). These agreements support, for instance, the selection of local leaders and community representatives for meeting and workshops forums where diverse members can raise concerns and influence decision-making processes in real time.

Local rules also support the establishment of management practices to cope with the impacts of seed harvesting in the plants and ecosystems (Meissen et al. 2015; Liu et al. 2019). Seed harvesting without damaging adult plants tends to have lower impacts than harvesting other plant’s parts, particularly for perennial plants (Schmidt et al. 2011). Species selection for seed collection must consider species abundance, distribution and conservation status. Importantly, rare, endemic and/or endangered species should not be prioritized for commercial seed collection programs to avoid impacts on native populations. In addition, seed suppliers should establish and observe the seed harvesting scale and frequency to allow natural regeneration of plant populations and food supply for granivores and frugivores (Nevill et al. 2018). In the Atlantic Forest in Brazil, collectors of the Vale do Ribeira Network agreed to not harvest at least half of the native seeds of the selected species. These collectors also undertake restoration projects in their communities to improve the seed source availability and the local environmental conditions.

Agreements and contracts should also be established between stakeholders outside the community groups to ensure their roles within the network. Although decentralized operations and power devolution are essential components to promote local engagement (Valette et al. 2020), the state has the vital role of enforcing laws and creating incentives to support local participation and benefits (Ribot et al. 2006). These processes are not a result of signed contracts but rely on the capacity to effectively negotiate a clear understanding and respect of the unique perspectives that shape the place-specific community organization.

### Incorporating local knowledge into technical development

Incorporating local knowledge and stimulating learning processes between local communities are crucial components of participatory seed programs (Baumann et al. 2020). Sharing experiences between community members, researchers and practitioners create opportunities to fully interconnect local experiences with scientific frameworks to co-design plural ways to operate supply systems (Kindt et al. 2006). A key advantage of prioritizing Indigenous participation is connecting to traditional lands of the community members who retain information and cultural values related to native species, vegetation and ecosystems (Brancalion et al. 2012). Beginning seed supply activities equipped with local knowledge is more effective, faster, and meaningful for promoting the development of local engagement than a standard ‘school room’ technical training approach (Campos-Filho et al. 2013).

The capabilities necessary for a fully functional community-based seed collection system are underpinned by how local communities perceive and determine the local needs, rather than offering a universal and specific set list of capabilities (Schlosberg and Carruthers 2010). When community members lead both planning and implementation of the learning processes to exchange experiences and innovate solutions, they are also building the social and political foundations for the development of an enduring and functional network. Through periodic meetings, workshops, and training courses, stakeholders can exchange and enrich knowledge sharing (Dawson et al. 2011). Such intercultural environments require the use of cross-culture educational material to effectively engage diverse groups, such as short documentaries, photo shoots and maps. The Gelganyem Seed Project in Australia promotes knowledge exchange events among Aboriginal groups and restoration practitioners to assist collectors in using their own experiences to identify species and collect the right type of seed in the right way. These training processes are designed based on the local community work plans and goals to encourage collectors to undertake the activities following their ways at their own pace.

Beyond seed production techniques, capacity-building assists local groups to strength skills and share experiences about business development, accounting, policy, and regulations. Building business models does depend upon seed collectors being able to manage their production costs (e.g., time allocated for each production activity and depreciation of equipment) to quantify seed production costs that ensure the long term viability of the commercial arrangements (Camhi et al. 2019). In central Brazil, collectors of the Association Cerrado de Pé have exchanged practices to design materials and mechanism for defining seed pricing. Collectors use a seed pricing formulation system based on a set of conditions applied for each species, and then during annual meetings, they make the seed trade agreements considering the seed production notes. This learning process among collectors and restoration practitioners has produced a significant amount of ecological data for 70 native species from savanna ecosystems.

### Improving practices to strengthen local engagement and benefits

Once seeds have left the community of local collectors and producers, and continue through the supply chain towards the restoration site, the original seed providers have little visibility of the fate of their seeds, and how ultimately their efforts impact restoration outcomes. Transparency and a proactive dialogue between seed producers and restoration practitioners are therefore critical to strengthen the native seed supply chain by (i) providing useful technical feedback to continually improve practices and efficiencies and (ii) increase motivation, engagement and commitment of the local community through positive feedback that locally sourced seed is ‘making a difference’. For example, if the seed success rate on a restoration site is low, but seed quality information is known, then other causes of failure can be investigated and resolved, such as timing of seeding, seeding depth, seeding density, site preparation, seeding time (Shaw et al. 2020).

Community participation in the restoration economy is required beyond native seed supply systems. Native seed production areas and plant nurseries, for example, offer abundant opportunities to increase the availability of high-quality plants for restoration, while strengthening economic returns (Nevill et al. 2018). In Western Australia, the partnership between different stakeholders and the Midwest Employment and Economic Development Aboriginal Corporation (MEEDAC) identified the role of Aboriginal leadership in implementing the first Australian Indigenous-owned native seed farm in Morawa for supplying local market needs in post-mining restoration. Despite the costly initial financial and infrastructural investments, seed production areas in the long run upscale supply systems and improve the economic outcomes for producers as a better alternative to wild seed collection (Nevill et al. 2018). In this way, community groups can be active participants in the long term ecological, human health, and economic benefits of rebuilding nature and restoring global ecosystems.

## Next steps

Community restoration networks focused on efficiency and revenue alone will not guarantee the long-term sustainable success of local seed production. We suggest that participation in seed supply requires full recognition of geographical, cultural, and social dynamics that transform structural power asymmetries into effective justice procedures for scaling up equitable restoration interventions. It is central to enshrining participation that builds visibility and recognises diverse groups within local communities (Schlosberg 2004). Beyond the local scale, it is essential that local-informs-global to ensure co-creation of policies and programs that are well-aligned with the reality and needs (Jalonen et al. 2017).

Seed programs are just the beginning of the shared journey towards community engagement linked to nature and natural environments. A transformative restoration economy to overcome structural inequalities during The Decade on Ecosystem Restoration must recognize that degraded lands and environmental impacts are often the result of historical socio-spatial asymmetries. Policies, funding support, and business investment for landscape restoration designed within national or international frameworks may not reflect the intentions and aspirations of local communities (Höhl et al. 2020). Community supply systems for restoration will only address the ongoing social and environmental inequalities if its instruments are applied as mechanisms to reshape the power dynamics among stakeholders executed in a respectful, open and informed way. This requires incorporating justice principles at the heart of restoration policies, funding, and actions through the participation of diverse community members to influence and shape multiple decision-making processes (Martin et al. 2020).
